# Blood-spinal cord barrier alterations in a mouse model of centrifugation-induced hypergravity

**DOI:** 10.3389/fphys.2025.1658389

**Published:** 2025-09-02

**Authors:** David Dubayle, Jean-Luc Morel, Sighild Lemarchant

**Affiliations:** ^1^ Paris Cité University, CNRS UMR8002, INCC, Paris, France; ^2^ Bordeaux University, CNRS UMR5287, INCIA, Bordeaux, France; ^3^ Axoltis Pharma, Lyon, France

**Keywords:** tight junctions, occludin, fibrinogen, blood-spinal cord barrier, hypergravity

## Abstract

Astronauts endure repetitive episodes of hypergravity (HG) during takeoff and landing of spaceflights, and also in space due to microgravity. Blood-brain barrier alterations and disruptions were recently reported in mice subjected to a short period of HG induced by centrifugation. In this study, we have evaluated if the blood-spinal cord barrier (BSCB) was also damaged by centrifugation-induced HG in mice. For that purpose, adult C57Bl/6J male mice were subjected to HG in a centrifuge at 2 g for 24 h, while control mice stayed in normogravity (n = 7-8 per group). Mice were sacrificed after centrifugation, and thoracic spinal cords collected for immunohistochemistry. Alterations of the BSCB were evaluated by measuring the immunoreactive areas of tight junction-associated proteins (claudin-5, occludin and zonula occludens-1 (ZO-1)) normalized to that of collagen IV-positive vessels. Additionally, the extravasation of a large blood-derived protein, fibrinogen, was quantified to determine if BSCB integrity was strongly impaired. Interestingly, a significant decrease in occludin level was measured in the spinal cord of HG 2 g mice compared to that of control 1 g mice (−28.6%, p = 0.0378), whereas claudin-5 (−20.6%, p = 0.2030) and ZO-1 (−19.6%, p = 0.3048) levels were not significantly affected. The decrease in occludin tight junction proteins was not accompanied by an extravasation of fibrinogen into the spinal cord parenchyma (p > 0.05). Overall, this study reports for the first time structural alterations of the BSCB in the context of hypergravity.

## 1 Introduction

The blood-brain barrier (BBB) is a dynamic interface between the bloodstream and the cerebral parenchyma, that maintains brain homeostasis by selectively regulating the transport of ions, nutrients, molecules and cells from the systemic circulation (Lacoste et al., 2025; Greene et al., 2019). Briefly, this interface is formed by a layer of microvascular endothelial cells surrounded by pericytes and astrocyte endfeet which are embedded in a parenchymal basement membrane. The paracellular selectivity and polarity of this semipermeable barrier is ensued by adherens, tight and gap junctions between neighboring microvascular endothelial cells. A similar barrier is also present in the spinal cord, and is known as the blood-spinal cord barrier (BSCB) (Bartanusz et al., 2011). Ultrastructural alterations of tight junctions (TJs) and the subsequent entry of circulatory molecules into the parenchyma have been reported in numerous pathologies and injuries affecting neural tissues (Lemarchant et al., 2025). These include notably Alzheimer’s disease (Xu et al., 2025a; Yue et al., 2024; Yamazaki et al., 2019), multiple sclerosis (Zierfuss et al., 2024; Oghabian et al., 2022; Leech et al., 2007) and Parkinson’s disease (Lau et al., 2024; Al-Bachari et al., 2020; Pienaar et al., 2015). BBB disruption is also encountered by healthy individuals following physiological changes induced by diverse external conditions such as high altitude (Lafuente et al., 2016) or diving (Sanchez-Villalobos et al., 2022). Similarly, hypergravity (HG) endured by astronauts might also alter the BBB (Dubayle et al., 2023). Indeed, we have recently reported subtle leakage of the BBB in the cortex and hippocampus of mice subjected to a 24-h centrifugation at 2 g to mimic HG (Dubayle et al., 2023). In the present study, we have evaluated if the BSCB was also damaged in this experimental model of HG generated by centrifugal acceleration. For that purpose, we measured the spinal levels of TJ proteins (claudin-5 and occludin), and of zonula occludens-1 (ZO-1), a membrane-associated protein that anchors TJ complexes to the cytoskeleton, in mice exposed to normal gravity (i.e., 1 g) or HG (2 g) for 24 h. Finally, we quantified the presence of a blood-derived protein (fibrinogen) into the spinal cord parenchyma.

## 2 Materials and methods

### 2.1 Animals

The experimental protocol has been approved by the French ministry of research and the local ethical committee of the PLEXAN platform (Faculty of Medicine, Université Jean Monnet Saint-Etienne). Fifteen (15) adult C57Bl/6J male mice of 8 weeks old obtained from Janvier Labs (Le Genest Saint Isle, France) were kept for housing and experiments took place within the animal resource centre located at PLEXAN where mice were tagged with tattoos for identification, and housed 3 per cage under standard conditions (22 °C ± 1 °C, humidity 55% ± 5%; day/night cycle 12 h/12 h) with an unlimited access to food and water. Mice were acclimated to their new environment for 3 days before the start of the experiments. A constant weight of food pellets (i.e., 125 g) was placed in the grid of each cage 48 h before centrifugation, and the remaining food pellets were weighed after the centrifugation to see if the centrifugation had an effect on food consumption.

### 2.2 Hypergravity

**FIGURE 1:**
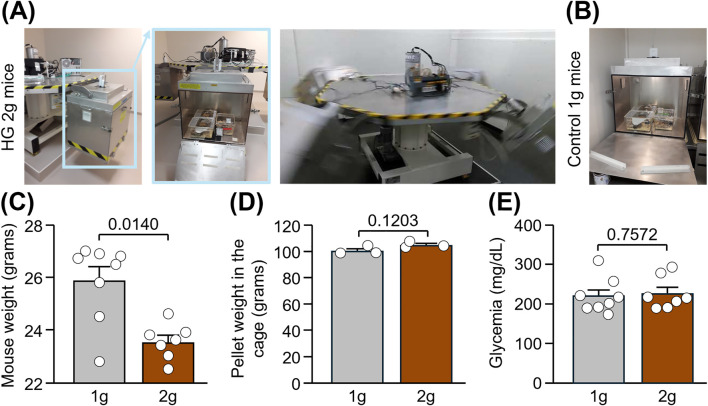
Body weight, food intake and glycemia in mice subjected to normogravity and hypergravity. **(A,B)** Centrifuge set-up made of 4 gondolas (indicated in blue in the pictures) in which adult male mice were subjected to HG at 2 g for 24 h **(A)** while control mice stayed at 1 g in an immobilized gondola for the same time **(B)**. **(C)** Mouse weight after centrifugation. Mann-Whitney test: *p < 0.05 HG 2 g vs. Control 1 g (n = 7-8 per group). **(D)** Average weight of pellets present in the grid of each of the 3 cages per group after centrifugation. Unpaired t-test: p > 0.05 (n = 3 per group). **(E)** Glycemia after centrifugation. Unpaired t-test: p > 0.05 (n = 7-8 per group).

We have previously shown a similar extravasation of immunoglobulin G (IgG) across the BBB after either short or long centrifugations at 2 g (i.e., for 24 h and 50 days, respectively) (Dubayle et al., 2023; Dubayle et al., 2020); for both practical and ethical reasons, we opted for the 24 h protocol for this study. Hypergravity above 2 g is associated with stress (Pulga et al., 2016; Guéguinou et al., 2012; Yuwaki and Okuno, 2004) and deleterious physiological effects (Guéguinou et al., 2012; Beraneck et al., 2012), which further emphasizes the relevance of 2 g intensity to our study. In our model, the serum level of corticosterone, a major stress hormone, did not increase in mice exposed to 2 g for 24 h compared to that of control littermates stayed in normogravity (Dubayle et al., 2023). Briefly, 3 mice per cage (cage size: 36 cm × 20 cm × 14 cm) were placed in the 4 gondolas (3 cages per gondola) of a centrifuge built by Comat (Toulouse, France). After habituation in the gondola, mice were subjected to HG at 2 g for 24 h (Figure 1A; n = 7). In parallel, control mice maintained in normogravity at 1 g were placed in cages located in a static position in the same room as the centrifuge and at the same time, in order to expose them to similar environmental conditions except for the centrifugation (Figure 1B; n = 8).

### 2.3 Sacrifice and preparation of spinal cord sections

Mice were anesthetized with intraperitoneal injections with xylazine (10 mg/kg; 6835444, Alcyon, Neuilly-sur-Seine, France) and ketamine (100 mg/kg; 6740407, Alcyon) before performing intracardiac perfusion of 15 mL phosphate-buffered saline (PBS 0.01 M, pH 7.4; P3813, Sigma-Aldrich, Saint-Louis, MO, United States) followed by 25 mL in a 10% formaline (HT501128, Sigma-Aldrich) solution to rinse the blood and to fix the tissues, respectively (flow of 5 mL/min). Glycemia was measured in the blood coming from the heart right before perfusion using a blood glucose meter (ACCU-CHEK, Roche, Basel, Switzerland), to see if the centrifugation had an effect on it. The mice were sacrificed within 3 h after stopping the centrifuge. The spinal cords were collected and the thoracic part of each spinal cord (T1-T3 levels) was isolated and post-fixed in the same formaline solution described above during 24 h at 2 °C–8 °C. They were then dehydrated in 70% ethanol and immersed consecutively in two baths of 90% ethanol, three baths of 100% ethanol and three baths of methylcyclohexane, before being embedded in wax (Tissue-Tek® Paraffin Wax TEK III; 4508, Sakura, Torrance, CA, United States) using an automated tissue processor (Leica, Wetzlar, Germany). Paraffin blocks were stocked at room temperature (RT) until further processing. Six 5-µm-coronal sections (100 µm apart of each other) from each thoracic spinal cord were cut on a microtome (Leica RM2255, Microm HM355S, Leica), collected on Superfrost + slides (J1800AMNZ, ThermoFisher Scientific, Waltham, MA, United States), dried during 1 h at 60 °C, and stored at RT until analysis.

### 2.4 Immunohistochemistry of TJ-associated proteins

Immunohistochemistry was performed using an automated staining system (Discovery XT, Roche) and associated reaction buffers containing CC1 (06414575001, Roche). Sections were washed and then exposed to primary antibodies for 1 h at RT with dilutions as follows: rabbit polyclonal anti-claudin-5 antibody (34-160, ThermoFisher Scientific; dilution 1:100), goat polyclonal anti-ZO-1 antibody (ab190085, AbCam, Cambridge, United Kingdom; dilution 1:100), rabbit monoclonal anti-occludin antibody (ab216327, AbCam; dilution 1:100) and goat polyclonal anti-collagen IV antibody (AB769, Sigma-Aldrich; dilution 1:50). Washed sections were then incubated for 1 h at RT with fluorescent Alexa fluor-conjugated secondary antibodies as follows: goat anti-rabbit AF555 antibody (A-21428, Invitrogen, Carlsbad, CA, United States; dilution 1:500) and donkey anti-goat AF647 antibody (A-21447, Invitrogen; dilution 1:500). Sections were then washed, stained with 300 nM DAPI (D21490, Molecular Probe, Eugene, OR, United States) and mounted on coverslips using Fluoromount (K024, CliniSciences, Nanterre, France). Negative controls for unspecific binding of the secondary antibodies were conducted in parallel sections following the same procedures described above, except the incubation in primary antibodies. Sections were imaged using 20× magnification on an Axioscan Z1 microscope (Zeiss, Oberkochen, Germany). Immunoreactive areas for claudin-5, occludin and collagen IV staining were quantified using QuPath software (version 0.4.3) using a threshold algorithm of the signal intensity in manually-segmented ventral horns of the six spinal cord sections. ZO-1 is ubiquitously expressed (Li et al., 2004), therefore only ZO-1 co-localized with claudin-5 (=vessel specific) was used to measure the immunoreactive area, as previously described (Greene et al., 2024). Immunoreactive areas of TJ-associated proteins were then normalized to that of collagen IV-positive vessels, and expressed as percentages of the control 1 g group.

### 2.5 Immunohistochemistry of fibrinogen

Prior to this immunohistochemistry, antigen retrieval was achieved by incubating sections in a Tris buffered saline (TBS)-ethylenediaminetetraacetic acid (EDTA) solution (GV804, Agilent, Santa Clara, CA, United States), for 30 min at 85 °C. After cooling down to reach RT, sections were washed and incubated in a blocking buffer containing 0.05% Triton X-100 (T8787, Merck, Rahway, NJ, United States) and 2% bovine serum albumin (BSA; A9647, Merck) for 1 h at RT. Sections were washed with TBS-Triton solution and then exposed to lectin coupled to Dylight-649 (DL-1178-1, Vector Laboratory, Newark, CA, United States; dilution 1:500) for 2 h at RT. Sections were then washed twice with TBS-Triton and TBS. Sections were then exposed to a rabbit polyclonal anti-fibrinogen antibody (ab34269, AbCam; dilution 1:1,000) overnight at RT. Sections were rinsed using the same procedure as after lectin labelling and incubated with a donkey anti-rabbit AF594 secondary antibody (A21207, ThermoFisher Scientific; dilution 1:500) for 2 h at RT. The nuclei were stained with Hoechst (94403, Merck; dilution 1:5000) and sections were then washed with TBS. Sections were then incubated in 0.1% black Soudan (199,664, Merck) for 5 min at RT to avoid background staining due to autofluorescence signal. Finally, sections were rinsed briefly in ethanol 70°, rehydrated in water during 1 min and mounted on coverslips using Fluoromount (00-4958-02, ThermoFisher Scientific). Sections were imaged using 20× magnification and extended focus mode (5 layers with step size of 1.4 µm) on a Pannoramic scanner II (3DHISTECH, Budapest, Hungary). Immunoreactive particles of fibrinogen were quantified using QuPath software (version 0.5.1) on three manually-designed ROI to select the ventral horn, grey matter and white matter on the 6 spinal cord sections per animal. Results are expressed as percentages of the control 1 g group.

### 2.6 Statistical analyses

All values are expressed as mean ± standard error of the mean. Statistical analyses were performed using GraphPad Prism software package 10.4.2. and using Unpaired t-test for graphs containing groups that passed the Shapiro-Wilk normality test. Otherwise, the Mann-Whitney test was applied. Pearson or Spearman test were used for correlations when groups passed the normality test or not, respectively. An alpha level of p < 0.05 was used to determine the significance in all the statistical tests.

## 3 Results

Adult C57Bl/6J male mice were subjected to hypergravity (HG) in a centrifuge at 2 g for 24 h, while control mice stayed in normogravity at 1 g (Figures 1A,B; n = 7-8/group). Mice were live-recorded in the centrifuge, and we did not observe abnormal jumping or freezing. However, we cannot exclude fine adaptations in static and dynamic parameters of locomotion, especially considering alterations of vestibular functions in this model (Beraneck et al., 2012). Right after centrifugation, a significant reduction in the weight of mice was measured in HG 2 g mice compared to control 1 g mice (Figure 1C: −2.4 g; Mann-Whitney test, p = 0.0140), despite no difference in food intake nor glycemia between groups (Figures 1D,E: Unpaired t tests, p = 0.1203 and p = 0.7572, respectively). Taken together, these result suggest a dysregulation of lipid metabolism induced by hypergravity and confirms previous findings showing a loss of weight in the same experimental model (Dubayle et al., 2023).

**FIGURE 2:**
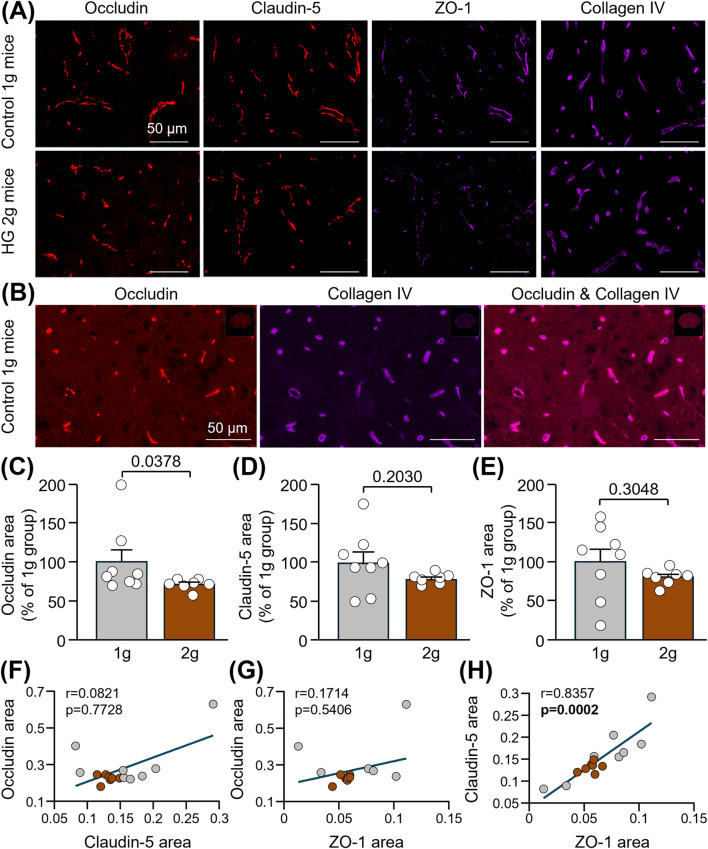
Reduction of TJ-associated proteins in the spinal cord of mice subjected to centrifugation-induced hypergravity. **(A)** Representative photomicrographs of occludin, claudin-5, ZO-1 and collagen IV in the ventral horn of the thoracic spinal cord of control 1 g mice and HG 2 g mice. Scale bars: 50 µm. **(B)** Images showing that, as expected, the TJ protein, occludin, co-localized with the vascular marker, collagen IV, in control mice. Scale bars: 50 µM. **(C,D)** Quantifications of occludin **(C)**, claudin-5 **(D)** and ZO-1 **(E)** immunoreactive areas normalized to that of collagen IV and expressed as percentages of the control 1 g group. Mann-Whitney test:*p < 0.05 for occludin, and Unpaired t-test: p > 0.05 for claudin-5 and ZO-1 (n = 7-8 per group). **(F–H)** Spearman correlations between occludin and claudin-5 (p > 0.05) **(F)**, occludin and ZO-1 (p > 0.05) **(G)** and claudin-5 and ZO-1 (p < 0.001) **(H)** (n = 15).

Mice were sacrificed shortly after centrifugation, and spinal cord slices extracted from the thoracic level were used for immunofluorescence of TJ-associated proteins (claudin-5, occludin and ZO-1) involved in the maintenance of BSCB integrity. A significant decrease in occludin level was measured in the ventral horn of the thoracic spinal cord of HG 2 g mice compared to that of control 1 g mice (Figures 2A–C: −28.6%; Mann-Whitney test, p = 0.0378). Conversely, no significant changes were observed for claudin-5 (Figures 2A,D: 20.6%; Unpaired t-test, p = 0.2030) and ZO-1 (Figures 2A,E: 19.6%; Unpaired t-test, p = 0.3048). Although no correlation was found between occludin and claudin-5 (Figure 2F: Spearman correlation, r = 0.0821, p = 0.7728) or ZO-1 (Figure 2G: Spearman correlation, r = 0.1714, p = 0.5406), reduced levels of claudin-5 were associated with lower levels of ZO-1 (Figure 2H: Spearman correlation, r = 0.8357, p = 0.0002), maybe as a result of the method used to quantify vessel-specific ZO-1 (please refer to the Materials and Methods).

**FIGURE 3:**
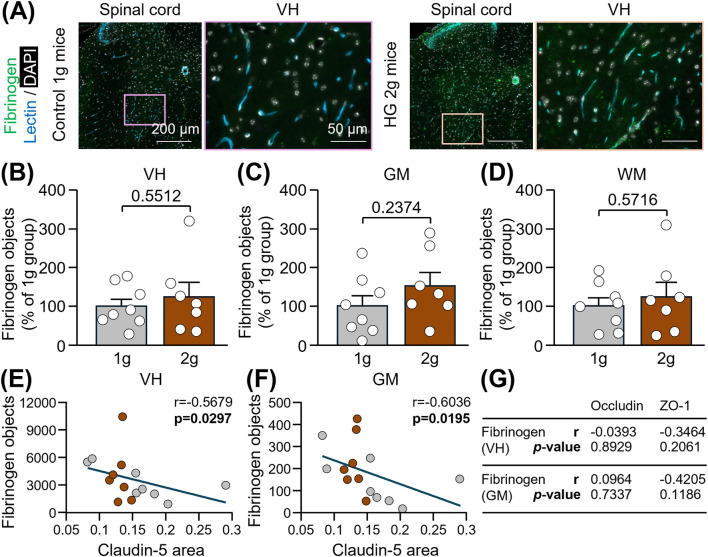
Evaluation of fibrinogen extravasation into the spinal cord of mice subjected to centrifugation-induced hypergravity. **(A)** Representative photomicrographs of fibrinogen in the thoracic spinal cord and a magnification of the ventral horn (VH) of control 1 g mice and HG 2 g mice. Scale bars: 200 and 50 µm respectively. **(B–D)** Corresponding quantifications of fibrinogen immunoreactive particles in the VH **(B)**, the total grey matter (GM; **(C)**) and the total white matter (WM; **(D)**). Unpaired t-test: p > 0.05 (n = 7-8 per group). **(E–F)** Spearman correlations between claudin-5 in the VH and fibrinogen, either in the VH **(E)** or in the total GM **(F)**: p < 0.05 (n = 15). **(G)** Table summarizing correlations between fibrinogen and occludin or ZO-1. Spearman correlations were used, except for the correlation between ZO-1 and fibrinogen in the GM for which the Pearson correlation was applied because the data were normally distributed in both groups.

Finally, we have examined if the reduction of TJ levels was accompanied by the presence of the blood-derived protein, fibrinogen, into the spinal cord parenchyma. No significant extravasation of fibrinogen was observed in 2 g mice compared to 1 g mice, neither in the ventral horn (Figures 3A,B: +24.3%; Unpaired t-test, p = 0.5512), nor in the total grey matter (Figures 3A,C: +53.2%; Unpaired t-test, p = 0.2374) or white matter (Figures 3A,D: +23.9%; Unpaired t-test, p = 0.5716). However, lower levels of claudin-5 were associated with an enhanced leakage of fibrinogen into the ventral horn (Figure 3E: Spearman correlation, r = −0.5679, p = 0.0297), as well as into the total grey matter (Figure 3F: Spearman correlation, r = −0.6036, p = 0.0195). All the correlations between fibrinogen and TJ-associated proteins are reported in Figure 3G.

## 4 Discussion

In the present study, we show for the first time structural alterations of the BSCB in a mouse model mimicking hypergravity. The levels of the endothelial TJ protein, occludin, were notably reduced by 30% in mice subjected to a centrifugation at 2 g for 24 h compared to that of mice stayed in normogravity (i.e., 1 g). These alterations of BSCB integrity did not result in a massive disruption of the barrier as suggested by similar levels of fibrinogen into the spinal cord parenchyma between groups.

Endothelial gene expression and functions are sensitive to gravity changes as previously reported *in vitro* and *in vivo* (Dubayle et al., 2023; Dubayle et al., 2020; Bauer et al., 2016; Costa-Almeida et al., 2016; De Cesari et al., 2020; Spisni et al., 2002; Szulcek et al., 2015; Kim and Kwon, 2018; Wehland et al., 2013). In our study, occludin was the only endothelial TJ-associated protein found to be reduced in the thoracic spinal cord of 2 g mice compared to 1 g mice. This is in accordance with the downregulation of *Ocln* gene expression in the hippocampus of 2 g mice (Dubayle et al., 2023) and of occludin protein levels in the choroid plexus of rats subjected to microgravity (Yang et al., 2024). In contrast, there was only a trend towards a decrease in the levels of the most expressed TJ protein at brain barriers (i.e., claudin-5) in the spinal cord of mice subjected to 2 g-gravity, which consolidates previous findings showing no changes in *Clnd5* gene expression in the hippocampus of mice subjected to the same experimental paradigm (Dubayle et al., 2023). Finally, ZO-1 is a scaffold protein that anchors TJ proteins to the cytoskeleton. In this study, ZO-1 levels were not significantly reduced in the spinal cord of mice subjected to a short centrifugation (i.e., 24 h) at 2 g, but ZO-1 protein levels were reported to be reduced in the cortex and hippocampus of mice after 35 days spent in the International Space Station (mostly under microgravity) (Mao et al., 2020). The short period of hypergravity versus the long period of microgravity could explain this difference in ZO-1 expression. Further studies are now warranted to determine if the effect of hypergravity on BSCB alterations of occludin or structural integrity of TJs by transmission electron microscopy are transient or persist over time.

The loss or discontinuity of TJs and related proteins, such as occludin, that seal the BSCB can lead to an abnormal leakage of blood components into the parenchyma and cause neuroinflammation and neuronal death (Lemarchant et al., 2025). This has been notably described in animal models of amyotrophic lateral sclerosis (Tang et al., 2025; Yoshikawa et al., 2022; Nicaise et al., 2009), degenerative cervical myelopathy (Schmidt et al., 2023), multiple sclerosis (Morgan et al., 2007) and spinal cord injury (Xu et al., 2025b; Wang et al., 2024; Zhou et al., 2023). Among these blood-derived proteins, fibrinogen, IgG and albumin are the most studied, yet they are large proteins and their permeability to the brain or spinal cord reflect a significant damage to brain barriers. Here, we did not observe an extravasation of fibrinogen (330 kDa) into the thoracic spinal cord in 2 g mice. Although the reduction of occludin levels was important, it did not lead to BSCB breakdown. However, it would be of interest to evaluate more subtle BSCB leakage by investigating the permeability of small fluorescent dextrans, delivered intravenously, into the spinal cord in mice subjected to hypergravity compared to that of control mice who stayed in normogravity. This experiment, if positive, would align with previous findings showing the passage of **1**) a 70-kDa FITC-dextran into the cortex and hippocampus of mice subjected to 2 g-gravity for 24 h, as opposed to larger exogenous tracers (Dubayle et al., 2023), and **2**) IgG (150 kDa) into the hippocampus of mice subjected to 2 g-gravity for 24 h and 50 days (Dubayle et al., 2020).

Astronauts and fighter pilots undergo short periods of hypergravity and endure reductions in cerebral blood flow that eventually leads to syncope (Burton and Smith, 2011; Blaber et al., 2011; Lyons et al., 2004). Hypergravity also triggers alterations in the cytoskeleton of human endothelial cells *in vitro* (Wehland et al., 2013), in the expression of genes encoding TJ proteins in mice (Dubayle et al., 2023) and in plasmatic biomarkers in humans (Verissimo Franca de Oliveira et al., 2025; Lang et al., 2024). It is therefore reasonable to believe that hypergravity elicits vascular stress responses that damage endothelial cells and *in fine* perturb homeostasis in the brain and spinal cord.

## 5 Conclusions/perspectives

Our results suggest alterations in BSCB integrity induced by a short period of hypergravity. Recent studies provided evidence that brain barriers disruption may be an early driver of neurodegeneration in ALS and frontotemporal dementia (Omar et al., 2025; Cheemala et al., 2025). Neurodegeneration in the spinal cord due to hypergravity could have neurological consequences such as motor and sensory deficits, or neuropathic pain. The possible impact of the loss of BSCB integrity on the appearance, progression and prognosis of neurological disorders should also be monitored closely.

Further studies are now warranted to better characterize and understand structural alterations and dysfunctions of the BBB and BSCB induced by gravity and radiation changes to which are exposed astronauts during spaceflights. As a first step, it would be advisable to investigate changes in gene expression of endothelial cells and in the integrity of brain barriers after long periods of hypergravity, microgravity and radiations. For example, induced pluripotent stem cells-derived brain barriers generated from skin fibroblasts or blood mononuclear cells of astronauts could be used to perform gene expression and protein assays of key structural elements of cerebral and spinal endothelial cells, such as claudin-5, occludin or ZO-1, and compare them to that of healthy controls who only experienced normogravity. It would be also informative to investigate the permeability of a gadolinium-based contrast agent in the brain and spinal cord of astronauts, prior and after spaceflights using dynamic contrast enhanced-magnetic resonance imaging, a technique commonly used for subtle detections of brain barriers leakage in patients with neurological disorders.

Finally, it can be argued that preventive or therapeutic agents aimed at protecting or repairing brain and spinal barriers may be beneficial to astronauts’ health.

## Data Availability

The original contributions presented in the study are included in the article/supplementary material, further inquiries can be directed to the corresponding authors.
